# A Stepwise Animated Video Modeling App With Token-Based Reinforcement for Toilet Training in Children With Autism Spectrum Disorder: Pilot Study Using Multiple-Baseline and Single-Case Designs

**DOI:** 10.2196/92147

**Published:** 2026-09-25

**Authors:** Inyoung Son, Younglae Kim, Yearin Kim, Jungmin Kim, Dongwon Kang, Sojung Lee, Jungsang Min, Bung-Nyun Kim

**Affiliations:** 1 Department of Research and Development, Emotiv Co., Ltd, Seoul, Republic of Korea; 2 Department of Child and Adolescent Psychiatry, Seoul National University Hospital, 101 Daehak-no, Chongno-gu, Seoul, 03080, Republic of Korea, 82 2-2072-364, 82 2-747-5774

**Keywords:** autism spectrum disorder, toilet training, mobile apps, video modeling, token-based reinforcement, activities of daily living, caregivers, pilot projects

## Abstract

**Background:**

Toilet training is a major challenge for children with autism spectrum disorder. While mobile health offers accessible support, few apps specifically address the complex chaining required for independent toileting routines.

**Objective:**

This pilot study evaluated the feasibility and preliminary effectiveness of a caregiver-mediated mobile app using stepwise animated video modeling with token-based reinforcement support for toilet training in young children with autism spectrum disorder.

**Methods:**

A multiple-baseline design across participants (urination training) and a single-case AB design (defecation training) were used. Ten children (aged 3‐6 years) were enrolled, and 4 (40%) were included in the final analyses. The tablet-based intervention guided a 16-step toileting routine using animation and token reinforcement. Primary outcomes were caregiver-recorded in-toilet elimination success (percentage) and routine completion (percentage). Secondary outcomes included standardized adaptive behavior scores (Korean Scales of Independent Behavior–Revised) and social validity.

**Results:**

Four children completed the study, whereas 6 were not included in the final analyses for heterogeneous reasons, including child engagement, caregiver implementation burden, and postenrollment ineligibility. Among completers, in-toilet elimination success improved substantially from baseline, reaching 100% for all participants at the limited 3-day follow-up observation window conducted 3 months after the intervention. Notably, one participant showed marked improvement from a stable 0% at baseline to 93.8% (30/32) of elimination events during the intervention. Routine completion also increased across all participants, suggesting improvements in independence. Caregivers reported favorable treatment adherence and social validity.

**Conclusions:**

The app showed preliminary feasibility and was associated with improvements in toileting outcomes among families who maintained engagement. However, the high attrition rate suggests that successful implementation may depend on child engagement readiness and caregiver resources. Future research should prioritize defining readiness criteria to identify families most likely to benefit from digital toileting interventions.

## Introduction

Autism spectrum disorder (ASD) is often accompanied by challenges in adaptive functioning and daily living routines, including difficulties with sequencing multistep self-care behaviors [1]. Among daily living skills, toileting is a major developmental milestone, yet many children with ASD experience persistent difficulties across the toileting routine—such as communicating need, clothing management, in-toilet elimination, and completing hygiene steps [2-4]. These difficulties can increase caregiver burden and interfere with participation in everyday routines at home and in early childhood settings (eg, day care or preschool), underscoring the need for feasible, family-centered toileting supports [5].

Behavioral toilet training packages—most notably those derived from rapid toilet training approaches—have been widely used for children with developmental disabilities, including ASD [4,6,7]. Although these approaches can increase in-toilet elimination, most published studies remain small sample sizes and report interventions and outcomes heterogeneously, which limits generalizability across children and settings [5]. In addition, many programs emphasize continence and scheduled routines while paying less attention to the broader toileting sequence required for independence (eg, clothing management, wiping, flushing, and handwashing) and to generalization across settings; some packages may also include aversive components that raise acceptability and ethical concerns [4,7,8].

In recent years, mobile health (mHealth) interventions for children with ASD have proliferated, leveraging the portability and accessibility of tablets and smartphones. However, recent systematic reviews indicate that most ASD-related mHealth apps focus on communication, social skills, or parent-mediated support rather than toileting-specific daily living routines [9,10]. Integrated digital tools that combine structured toileting routines, caregiver-mediated implementation, stepwise video modeling, reinforcement support, and home-based toileting data recording remain limited [11].

Video modeling is an evidence-based instructional strategy in which individuals observe a model performing a target behavior and then practice the same behavior, which can reduce verbal demands and leverage visual learning strengths often reported in ASD [12-14]. Meta-analytic and experimental studies support video modeling for teaching functional skills to children with ASD [14-16]. In toileting, video modeling has shown promise for teaching parts of the toileting routine [17,18]. To address challenges in depicting sensitive behaviors (eg, in-toilet urination or defecation), some studies have incorporated animation to visually represent the full toileting sequence [19,20]. However, for multistep routines, video-based instruction can still be constrained by attention demands and prerequisite learning skills when sequences are long or complex; segmented and stepwise formats have therefore been proposed to support acquisition of response chains [21].

Because toileting requires repeated practice over extended periods, sustaining children’s participation and caregiver implementation is a practical challenge. In ASD-focused digital interventions, game-related features (eg, simple token collection, visual rewards, or optional mini games) have been used to support engagement and repeated practice, although evidence remains limited and mixed for daily living skills [22,23]. Technology-assisted delivery may also help standardize modeling, provide in-the-moment prompts, and support caregiver-mediated implementation in naturalistic home settings.

Accordingly, this pilot study describes the development and preliminary evaluation of a tablet-based toileting program that delivers brief, stepwise animated video models with simple interactive prompts, caregiver-entered performance feedback, and a token-based reinforcement system (digital stickers exchanged for preferred reinforcers, with optional mini games). Using single-case experimental designs (a multiple-baseline design across participants for urination training and a single-case AB design for defecation training) with 4 preschool-aged children with ASD, we explored feasibility and preliminary patterns of change in toileting routine completion and independent toileting performance over time.

## Methods

### Study Design

This pilot study used single-case experimental methods. A multiple-baseline design across participants was applied to evaluate the program for urination (participants T06, T09, and T10), and a single-case design with baseline, intervention, and follow-up phases was used for defecation training (participant T05).

### Ethical Considerations

This study was approved by the Public Institutional Review Board designated by the Ministry of Health and Welfare of South Korea (P01-202308-01-056). All procedures were conducted in accordance with the Declaration of Helsinki. Written informed consent was obtained from all parents or legal guardians prior to participation.

### Participants and Recruitment

**Figure 1.**
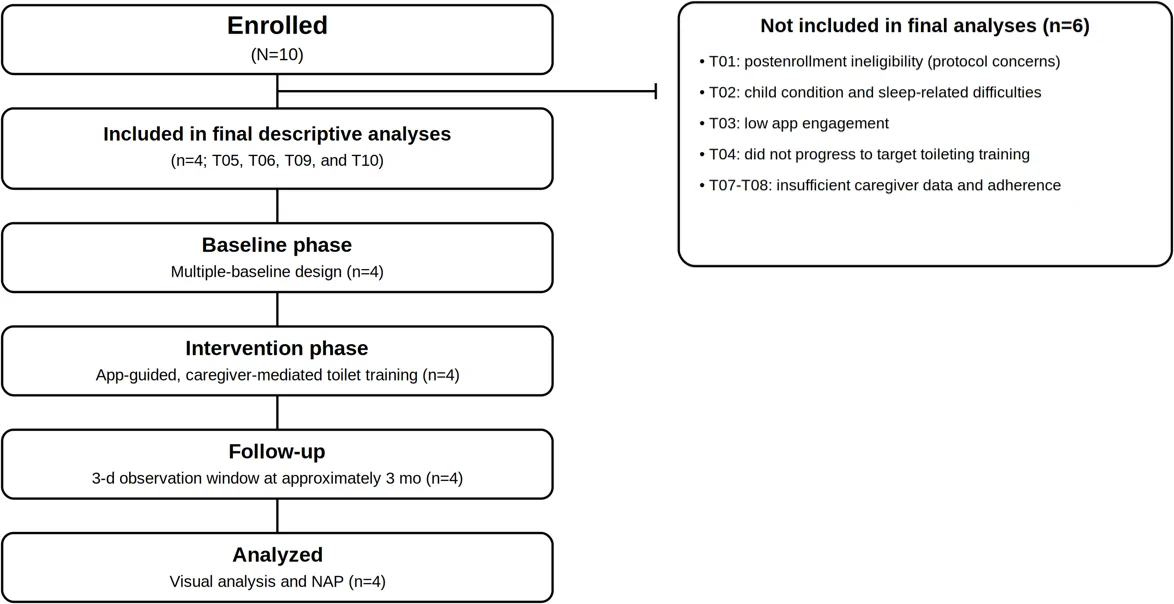
Participant flow. NAP: nonoverlap of all pairs.

Children were recruited through an online community for caregivers of children with developmental disabilities. Children were eligible if they were aged 3 to 6 years and had either a clinical diagnosis of ASD by a pediatrician or an ongoing diagnostic evaluation for suspected ASD at enrollment. Children were excluded if they were receiving ongoing toilet training outside the study protocol at enrollment. Eligibility additionally required persistent toileting difficulties (eg, elimination outside the toilet, no spontaneous toilet visits, and/or prior attempts with continued problems); no participation in a structured toileting intervention within the 4 weeks prior to enrollment; and the ability to understand simple instructions, with no reported vision or hearing impairments. Formal standardized measures of imitation ability or receptive language were not collected at screening. Withdrawal and discontinuation criteria were defined a priori and applied consistently: participation could be discontinued if (1) unexpected adverse responses or safety concerns arose, (2) repeated refusal or low engagement prevented feasible delivery of the training procedures and/or adequate recording, (3) a new structured toileting intervention was initiated during the study period, or (4) the caregiver withdrew consent. Four children (T05, T06, T09, and T10; n=2 girls) met the prespecified analytic inclusion criterion (≥5 caregiver-reported app-guided training sessions) and were included in the final analyses. Participant flow is shown in Figure 1.

### Setting and Materials

The intervention was delivered via a mobile app compatible with smartphones and tablets and was implemented by caregivers in naturalistic settings, primarily the children’s home bathrooms. To standardize access and screen size across families, caregivers were provided with a 10.4-inch Samsung Galaxy tablet with the app preinstalled. Caregivers were asked not to introduce new toileting interventions during the study and to keep the children’s usual toileting routines and concurrent therapies as consistent as feasible across phases.

### App Development and Design Rationale

#### Overview

The app was developed to support toilet training for young children with ASD by integrating video modeling with applied behavior analytic principles to facilitate chaining of a multistep toileting routine [24,25]. The toileting routine was operationalized as a 16-step task analysis (Textbox 1) developed in consultation with a board-certified behavior analyst at Seoul National University Hospital. Segmenting self-care routines into teachable units was intended to support children who commonly experience difficulty with sequencing and adaptive functioning [1].

Textbox 1. **The 16-step toileting routine.**

- Step 1: requesting the bathroom
- Step 2: turning on the light
- Step 3: entering the bathroom
- Step 4: checking the toilet lid
- Step 5: pulling down pants
- Step 6: pulling down underwear
- Step 7: sitting on the toilet
- Step 8: eliminating in the toilet (urination and/or defecation)
- Step 9: wiping with toilet paper
- Step 10: putting on underwear
- Step 11: putting on pants
- Step 12: flushing the toilet
- Step 13: washing hands
- Step 14: drying hands with a towel
- Step 15: leaving the bathroom
- Step 16: turning off the light

#### Rationale for Animation and Character-Based Modeling

The modeling content was implemented as animation rather than live-action video to reduce potential discomfort and privacy concerns associated with depicting sensitive toileting-related behaviors (eg, wiping) using human actors and to present a child-friendly character that may be easier for young children to attend to and imitate. Video modeling aligns with learning characteristics commonly described in ASD by reducing verbal demands and emphasizing visual information [13,14]. Prior toileting-focused interventions have incorporated animation within video modeling to represent sensitive behaviors while preserving a complete routine sequence [19,20].

#### Reward Features and Reinforcement

The app incorporated a reward structure consistent with token-based reinforcement [24]. Digital sticker tokens were delivered contingent on successful completion of targeted steps. Tokens were accumulated and exchanged using a fixed rule (10 tokens→immediate reward). Reward options could include in-app content (eg, mini games or customization and decoration features) and/or caregiver-selected preferred items or activities available in the bathroom area, allowing for individualized reinforcement while keeping the exchange rule consistent. No punishment or aversive procedures were used.

### Intervention: Stepwise Animated Video Modeling App

#### Overview

**Figure 2.**
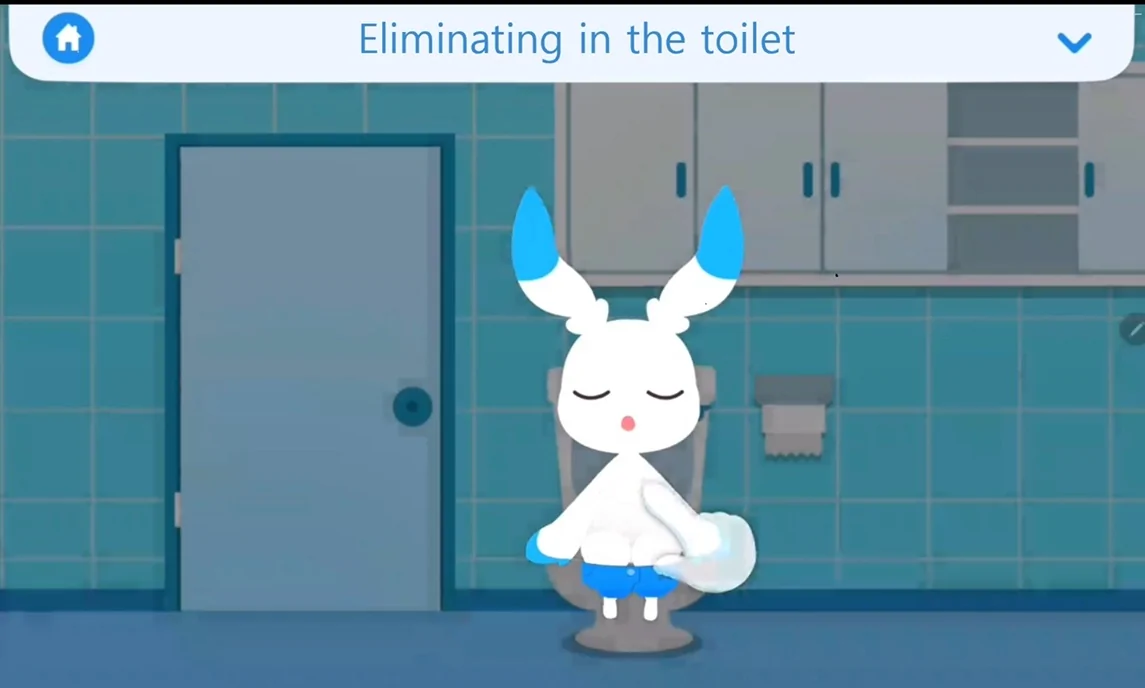
User interface of the stepwise animated video modeling.

The toileting routine was structured as a chained sequence of 16 steps. Caregivers selected target steps for training sessions based on the children’s needs. All step-specific animated models featured a single rabbit character (Rudy) to provide consistent visual cues across steps and repeated practice opportunities. The app interface is shown in Figure 2.

#### Session Flow and Feedback

Each training session followed a standardized flow for the selected steps: (1) the child watched the step-specific animated model, (2) the app prompted imitation (“Do what I did!”), (3) a pop-up rating question appeared (eg, “Did your child copy well?”) with 2 response options (“Good job” or “Not really”), and (4) feedback was delivered based on the caregiver’s response. If “Good job” was selected, the caregiver provided brief praise, and the app displayed a reward animation and delivered a digital sticker token that the child could drag and attach to a collection area. If “Not really” was selected, no reward feedback was provided and the modeling segment was replayed, allowing up to 2 additional attempts for that step.

#### Token Exchange Rule and Session Schedule

Sticker tokens served as digital tokens. Once the child accumulated 10 tokens, the preselected reward was delivered immediately in the bathroom area. During the intervention phase, caregivers were instructed to conduct at least one app-guided training session per day; training frequency could be individualized (eg, once daily vs 3 times daily) and was kept stable within each child once the intervention began.

### Outcomes and Measures

Outcomes were grouped into 4 domains to describe elimination performance, toileting routine performance, caregiver-reported functioning, and implementation.

#### Caregiver-Recorded Toileting Logs

Caregivers recorded toileting behavior using an online tracking sheet (Google Forms) provided by the research team. Logs were completed whenever the child urinated or defecated or participated in toileting practice and included date and time, location, elimination type (urination or defecation), in-toilet success, and step-level items aligned with the 16-step routine, with optional comments. Because children attended day care, kindergarten, or school, caregivers primarily recorded events occurring when the children were under caregiver supervision; events outside caregiver observation may not have been captured.

#### Elimination Outcome: In-Toilet Elimination Success

In-toilet elimination was defined as urination or defecation occurring into the toilet bowl. Phase-level success rates were calculated separately for urination and defecation as (number of in-toilet elimination events/total elimination events recorded) × 100. For target-specific elimination outcomes, denominators were based on the elimination type selected in the caregiver log. For urination training participants, events categorized as urination or mixed urination and defecation episodes were included in the urination denominator. Mixed events were coded as in-toilet successes only when the caregiver marked the episode as successful. Records in which urination was mentioned only in free-text comments but not selected as the elimination type were not counted. Practice episodes without elimination, nontarget elimination events, and entries with insufficient information were excluded from the corresponding outcome denominator.

#### Toileting Routine Performance Outcome: Daily Completion of the 16-Step Routine

Caregivers recorded step-level completion aligned with the 16-step routine for each recorded log entry (including practice episodes regardless of whether elimination occurred). Daily routine completion was calculated as (sum of successfully completed steps recorded that day/total step opportunities recorded that day) × 100. Total step opportunities were defined by the number of recorded log entries for that day multiplied by 16 steps (based on the standardized routine). For example, if 2 log entries were recorded on a given day, the total step opportunities were 32; if 24 steps were completed across those entries, the daily completion for that day was 75%. Given variability in the number of recorded entries per day, day-level percentages were interpreted primarily by patterns over time (eg, trends) rather than isolated single-day values. Because elimination cannot be ethically controlled, routine performance was evaluated separately from elimination outcomes. Step 8 (eliminating in the toilet) was coded as completed only when urination and/or defecation occurred. Practice entries in which elimination did not occur were retained for routine performance analyses, but step 8 was coded as not completed for those entries; elimination outcomes were captured only through the event-based in-toilet success metric.

#### Toilet Use Method (Initiation and Assistance Categories)

For each log entry, caregivers selected one mutually exclusive category describing how the toileting episode occurred: caregiver prompting, child communication or indication of toileting need, independent toilet use, or accident (elimination outside the toilet). Phase-level percentages were summarized descriptively for each participant.

#### Caregiver-Reported Outcomes (Standardized Measures)

Caregivers completed standardized questionnaires online at prespecified time points.

The Korean version of the Gilliam Autism Rating Scale–Second Edition (K-GARS-2) is a caregiver, teacher, or clinician–rated autism screening measure for individuals aged 3 to 22 years [26,27]. It includes 42 items across 3 subscales—stereotyped behaviors, communication, and social interaction—yielding standardized scores.

The Korean Scales of Independent Behavior–Revised (K-SIB-R) is a standardized, norm-referenced measure of adaptive functioning [28]. In this study, we summarized (1) toileting age-equivalent scores (years and months) and (2) Personal Self-Care W scores. In addition, to contextualize change relative to age expectations, we expressed Personal Self-Care W scores as deviation from age-based norms in SD units using normative values from the K-SIB-R manual; normative mean and SD values are not reported in this manuscript.

The Behavior Rating Inventory of Executive Function–Preschool Version (BRIEF-P) is a caregiver-rated measure of executive functioning for preschool children [29]. It includes 63 items across 5 clinical scales (inhibit, shift, emotional control, working memory, and plan or organize), with higher scores indicating greater executive function difficulties.

The Korean Parenting Stress Index, Fourth Edition, Short Form (K-PSI-4-SF) assesses parenting stress among parents of children aged 1 to 12 years [30]. It comprises 3 subscales—parental distress, parent-child dysfunctional interaction, and difficult child—and yields a total stress score, with higher scores indicating greater parenting stress.

### Treatment Fidelity

Treatment fidelity was evaluated using a 7-item checklist developed for this study based on a prior Korean intervention study using a social validity measure [31]. Caregivers completed the checklist on 5 occasions per participant during the intervention period, rated adherence on a 5-point scale, and reported deviations or modifications. The items assessed were (1) preparation of the training environment, (2) arrangement of materials or reinforcers, (3) caregiver instruction to the child, (4) correct software use, (5) waiting for the child’s response, (6) delivery of reinforcement for correct responding, and (7) appropriate responding to incorrect behavior (eg, replay without reinforcement). Fidelity was calculated as (acquired score/total possible score) × 100. The treatment fidelity checklist items can be found in Table S1 in Multimedia Appendix 1.

### Social Validity

Social validity was assessed after the intervention using a caregiver questionnaire (9 items, 5-point Likert scale). Items assessed perceived effectiveness, acceptability, safety, feasibility, and overall satisfaction with the toileting program. The questionnaire was developed and refined based on a prior Korean intervention study reporting treatment fidelity procedures [31].

### Procedure and Study Phases

No parallel control group was included in this study; instead, experimental control was supported by the staggered introduction of the intervention across participants.

Caregivers received study information and completed informed consent; the device was distributed; caregivers received 1:1 training on app use and home implementation, including operational definitions for caregiver recording and completion of the Google Forms–based logs; and preintervention questionnaires and recording instructions were completed. During the study, the research team reviewed submitted records weekly and followed up with caregivers via telephone when needed to clarify missing or ambiguous entries.

At baseline, no app-guided training was conducted. Children followed usual toileting routines, and caregivers recorded toileting events and step-level items. Baseline duration was staggered across participants (minimum of 5 days).

During the intervention, caregivers implemented app-guided sessions daily (minimum once per day; the individualized frequency was kept stable within each child) and continued recording. The intervention was introduced sequentially across participants; a participant began the intervention when the preceding participant met an a priori decision rule (mean routine performance across 3 consecutive intervention days exceeding the baseline mean by ≥15 percentage points). The intervention phase ended when the mastery criterion (≥80% for 3 consecutive sessions or days) was met or when the discontinue criterion was met (no meaningful improvement over 15 sessions).

Caregivers completed questionnaires and implementation measures at the end of the intervention period, and a follow-up was conducted 3 months after the intervention and consisted of 3 recording days using the same logs; the app was not used during follow-up.

### Data Analysis

Descriptive analyses other than nonoverlap of all pairs (NAP) were conducted using R (version 4.3.1; R Foundation for Statistical Computing). Outcomes were summarized descriptively using visual analysis and phase comparisons. No confirmatory hypothesis testing was performed. To supplement visual analysis of single-case data, NAP [32] was computed using Python (version 3.13.5; Python Software Foundation) for each participant as a descriptive nonoverlap index for daily toileting routine completion. Follow-up days were not included in these calculations. For K-SIB-R Personal Self-Care W scores, SD unit deviation from age norms was computed descriptively as (W − age norm mean)/(age norm SD) using manual-based norms.

## Results

### Participant Flow and Available Data

A total of 10 children were enrolled. Four children (T05, T06, T09, and T10; n=2 girls) met the prespecified analytic inclusion criterion (≥5 caregiver-reported app-guided training sessions) and were included in the final analyses. Reasons for noncompletion or exclusion from the final analytic sample are summarized in Table S2 in Multimedia Appendix 1. Across phases, the number of caregiver log entries was as follows: n=10 at baseline and n=32 during the intervention for T05, n=42 at baseline and n=90 during the intervention for T06, n=51 at baseline and n=141 during the intervention for T09, and n=32 at baseline and n=144 during the intervention for T10. Follow-up consisted of 3 recording days, during which caregiver log entries were recorded for T05 (n=6), T06 (n=9), T09 (n=14), and T10 (n=14). Participant characteristics and baseline toileting profiles are shown in Table 1.

**Table 1.** Baseline characteristics of participants included in the final analyses.

Characteristics	T05	T06	T09	T10
Age	4 years, 3 months	5 years, 3 months	4 years, 6 months	6 years, 11 months
Sex	Female	Male	Male	Female
ASD^a^ diagnostic status	Confirmed	Confirmed	Clinical ASD concern; diagnosis pending	Confirmed
Relevant comorbidity	None reported	None reported	None reported	Intellectual disability
K-GARS-2^b^	100	79	94	<69
Language level	Spontaneous 2‐ to 3-word phrases	Several spontaneous words (eg, “diaper,” “take off,” “pee,” and “wet wipes”)	Spontaneous 2‐ to 3-word phrases for requests	Several spontaneous words (eg, “water,” “cup,” “mom,” and “dad”)
Digital device use	Able to use simple controls	Able to use tablet apps for watching videos at home	Able to select desired items with setup assistance	Able to activate a smart device and open desired apps
Training target	Defecation	Seated urination	Seated urination	Toileting request+seated urination
Baseline toileting profile	Defecated while squatting behind a curtain in the bedroom wearing underwear	Had urine accidents in clothing and walked around undressed afterward	Urinated in the toilet when assisted by the caregiver but had no independent toileting behavior	Self-initiated toilet attempts at home but had accidents outside the home without communication

a ASD: autism spectrum disorder.

b K-GARS-2: Korean version of the Gilliam Autism Rating Scale–Second Edition.

### In-Toilet Elimination Success

Table 2 summarizes the phase-level percentage of elimination events that occurred in the toilet bowl (in-toilet elimination). Because Table 2 reports target-specific elimination outcomes, the denominators in Table 2 differ from the total number of caregiver log entries reported above. Baseline in-toilet elimination success varied across participants (0%‐23/27, 85.2%). During the intervention, all participants showed higher in-toilet elimination success than at baseline, and all participants showed 100% in-toilet elimination based on the available events during the limited 3-day follow-up observation window.

**Table 2.** Target-specific in-toilet elimination success across study phases. Percentages were calculated using target-specific elimination events as denominators. Practice episodes, nontarget elimination events, and entries with insufficient information were excluded.

Participant IDs	Target outcome	Baseline, n/N (%)	Intervention, n/N (%)	Follow-up, n/N (%)
T06	Urination	10/35 (28.6)	62/84 (73.8)	8/8 (100)
T10	Urination	23/27 (85.2)	115/123 (93.5)	12/12 (100)
T09	Urination	37/45 (82.2)	131/134 (97.8)	13/13 (100)
T05	Defecation	0 (0)	30/32 (93.8)	6/6 (100)

For T06, in-toilet elimination increased from 28.6% (10/35) at baseline to 73.8% (62/84) during the intervention (+45.24 percentage points; Table 2). Follow-up in-toilet elimination success was 100% (8/8) based on the available follow-up events (limited 3-day observation window).

For T10, baseline in-toilet elimination was 85.2% (23/27) and increased to 93.5% (115/123) during the intervention (+8.31 percentage points; Table 2). Follow-up in-toilet elimination success was 100% (12/12) based on the available follow-up events (limited 3-day observation window).

For T09, baseline in-toilet elimination was 82.2% (37/45) and increased to 97.8% (131/134) during the intervention (+15.54 percentage points; Table 2). Follow-up in-toilet elimination success was 100% (13/13) based on the available follow-up events (limited 3-day observation window).

For T05, baseline in-toilet elimination was 0% and increased to 93.8% (30/32) during the intervention (+93.75 percentage points; Table 2). Follow-up in-toilet elimination success was 100% (6/6) based on the available follow-up events (limited 3-day observation window).

It should be noted that percentages were calculated using target-specific elimination events as denominators. Practice episodes, nontarget elimination events, and entries with insufficient information were excluded.

### Toileting Routine Performance: Daily Completion of the 16-Step Routine

**Figure 3.**
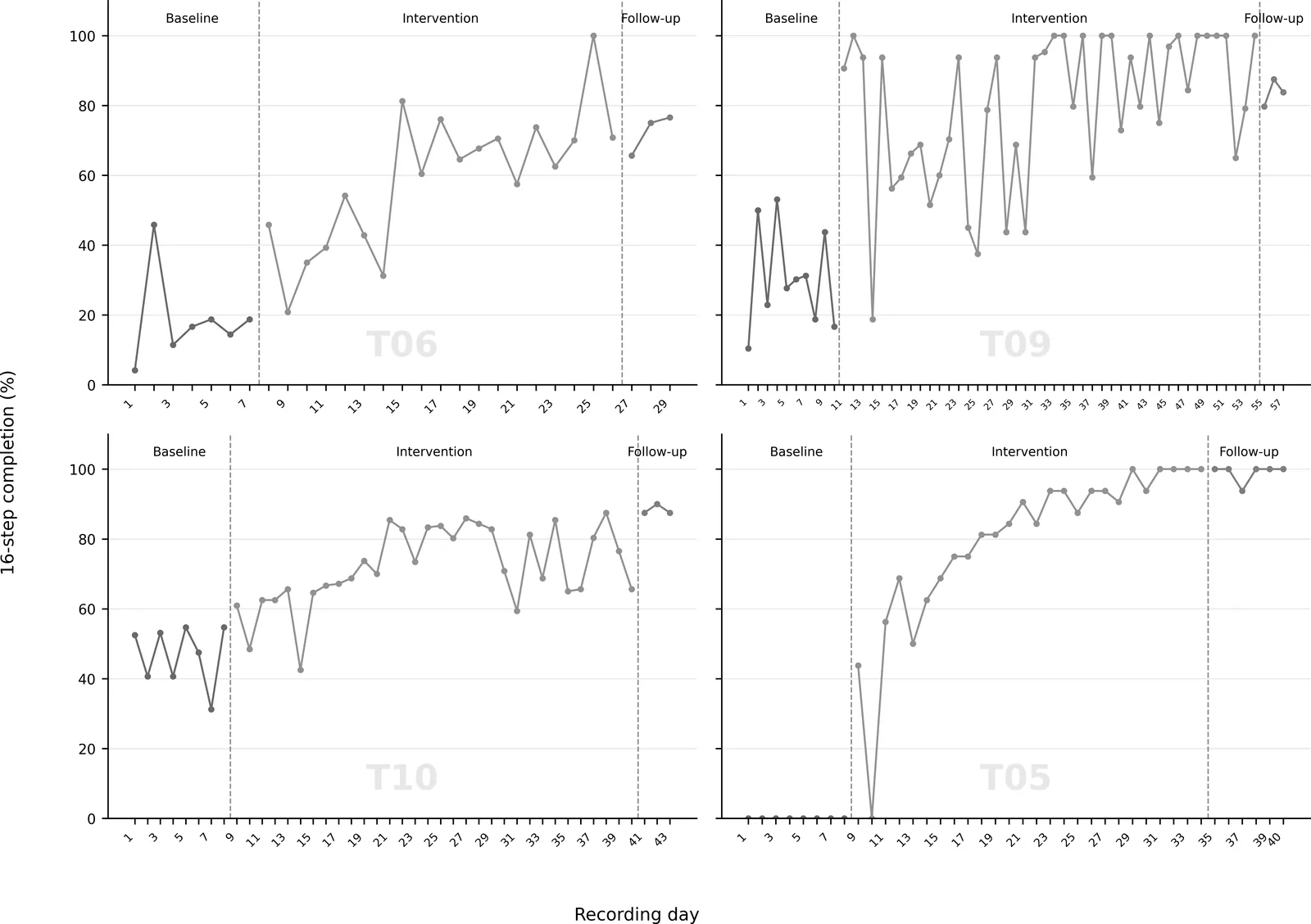
Day-level completion of the 16-step toileting routine across baseline, intervention, and follow-up phases. Each data point represents the mean percentage of completed steps across all caregiver-recorded entries within a recording day. The x-axis represents the sequence of recording days with available caregiver-recorded data and does not reflect the actual calendar interval between phases. Vertical dashed lines indicate phase transitions. Follow-up observations were collected approximately 3 months after the intervention phase, although the exact calendar interval varied across participants.

Daily toileting routine step completion (percentage) for all 4 participants is shown in Figure 3. Because the number of recorded log entries varied by day, day-level percentages were interpreted primarily by patterns over time (eg, level and trend). Visual inspection also indicated differences in the immediacy of effect: routine completion for T09 exceeded all baseline values from the first intervention days, and T05 increased from a stable 0% at baseline on the first intervention day, indicating relatively immediate changes in level, whereas T06 and T10 showed more gradual improvements across the intervention phase.

T06 showed a low and stable baseline followed by a clear upward shift and increasing trend after intervention onset, reaching sustained high performance later in the intervention phase and remaining elevated during follow-up.

T10 showed moderate baseline performance with variability; after intervention introduction, routine completion increased overall with intermittent dips, and follow-up values remained within the higher range observed late in the intervention.

T09 showed baseline variability; during the intervention, routine completion continued to fluctuate but with an overall upward pattern and repeated high-performance days later in the intervention. Follow-up values remained higher than baseline.

T05 showed 0% routine completion during baseline and increasing routine completion during the intervention, with high performance later in training and elevated values during follow-up.

Supplementary nonoverlap analyses using day-level 16-step completion percentages indicated substantial phase separation across participants, with NAP values ranging from 0.9580 to 0.9808 (Table 3). This index complemented the visual analysis by quantifying the degree of nonoverlap between the baseline and intervention phases.

**Table 3.** Nonoverlap indexes for day-level 16-step toileting routine completion^a^.

Participant IDs	Baseline days used, n	Intervention days used, n	Baseline daily 16-step completion (%)	Intervention daily 16-step completion (%)	NAP^b^
T05	8	26	0.00	79.57	0.9808
T06	7	19	18.58	59.18	0.9586
T09	10	44	30.48	79.76	0.9580
T10	8	32	46.88	71.93	0.9648

a “Days used” refers to recording days with available step completion data. Follow-up days were not included in nonoverlap analyses. T05 was evaluated in an AB design without staggered replication across participants; indexes for T05 are therefore descriptive only.

b NAP: nonoverlap of all pairs.

### Toileting Method (Initiation and Assistance Categories)

Phase-level distributions of toileting method categories (independent toilet use, caregiver prompting, child communication or indication, and accident) can be found in Table S3 in Multimedia Appendix 1. Overall, accidents decreased for T05, T06, and T09 during the intervention and were not observed at follow-up for any participant. Independent toilet use increased prominently for T05 and T06. Child communication or indication was observed primarily for T09 and T10 during intervention and follow-up, whereas caregiver prompting remained common, particularly for T09.

### Standardized Measures

Baseline characteristics, including K-GARS-2 scores, are shown in Table 1. K-SIB-R outcomes are summarized in Table 4.

**Table 4.** Korean Scales of Independent Behavior–Revised toileting age-equivalent (AE) scores and Personal Self-Care W scores (with norm-referenced deviation in SD units, which were computed descriptively using manual-based age norms as [W − age norm mean]/[age norm SD]; normative mean and SD values are not reported).

Participant IDs	Chronological age	Toileting AE		Personal Self-Care W, SD units			Norm-referenced deviation, SD units	
		Before	After	Before	After	ΔW	Before	After
T06	5 y, 3 mo	2 y, 2 mo	3 y, 7 mo	467	482	+15	−3.11	−1.08
T10	6 y, 11 mo	1 y, 11 mo	2 y, 7 mo	465	474	+9	−3.73	−2.65
T09	4 y, 6 mo	1 y, 11 mo	2 y, 7 mo	465	474	+9	−3.53	−1.99
T05	4 y, 3 mo	2 y, 8 mo	4 y, 0 mo	476	484	+8	−1.64	−0.27

On the K-SIB-R Personal Self-Care domain, age-equivalent scores increased from before to after the intervention. To contextualize change relative to age expectations, Personal Self-Care W scores increased for all participants (ΔW=+8 to +15), and deviation from age-based normative means decreased (Table 4). Notably, T06 moved from approximately 3 SDs below the age norm at baseline to approximately 1 SD below after the intervention, and T05 moved to within 1 SD of the age norm after the intervention. Despite improvement, some participants remained at 2 or more SDs below age norms after the intervention (eg, T10).

Exploratory changes in BRIEF-P subscale scores and K-PSI-4-SF percentile ranks can be found in Table S2 in Multimedia Appendix 1.

### Implementation Outcomes: Treatment Fidelity and Social Validity

Treatment fidelity scores, calculated from caregiver-completed fidelity checklists (5 administrations per participant), indicated favorable adherence to intervention procedures. Social validity ratings (Table 5) indicated favorable caregiver perceptions of acceptability, safety, and feasibility and overall satisfaction with the program.

**Table 5.** Mean scores on the social validity questionnaire from 4 caregivers of participants.

	Mean scores
Total	4.4
Understanding	4.75
Convenience	4.5
Reuse intention	4.25
Recommendation intention	4.75
Children’s immersion	4.5
Safety	4.75
Effectiveness	4.25
Expression increase	4.25
Hygiene independence	4

## Discussion

### Principal Findings

In this pilot study, a caregiver-implemented mobile app that combined stepwise animated video modeling with a structured reward system was associated with improvements in toileting behaviors among young children with ASD. Across participants, the proportion of elimination events occurring into the toilet increased during the intervention and showed 100% in-toilet elimination during the limited 3-day follow-up observation window conducted 3 months after the intervention. In parallel, completion of the 16-step toileting routine improved over time, suggesting gains in broader toileting-related self-care behaviors beyond voiding alone (eg, undressing, flushing, and hygiene steps). Caregivers also reported favorable adherence and favorable perceptions of acceptability and safety, supporting feasibility of home implementation for families who engaged with the app.

A key finding of this pilot study relates to the feasibility boundaries of caregiver-mediated digital interventions. We observed a high attrition rate, with 60% (6/10) of enrolled families discontinuing participation or being excluded from analysis for heterogeneous reasons, including child condition or sleep-related difficulties, low tablet or app engagement, inability to progress beyond toilet sitting readiness, caregiver recording or implementation nonadherence, and postenrollment ineligibility (Table S1 in Multimedia Appendix 1). The high attrition rate represents a major limitation of the present study but also provides useful feasibility information for future research. The pattern of discontinuation suggests that successful implementation of this app requires specific child prerequisites (eg, basic imitation skills and tolerance for tablet interaction) and sufficient parental resources to facilitate the initial sessions. This aligns with broader findings in eHealth research, where open trials often reveal high attrition, highlighting the need to tailor inclusion criteria to the families most likely to benefit [33]. Thus, the findings suggest that the intervention may be beneficial for families able to sustain engagement, but broader applicability requires further study with clearer inclusion criteria. Toileting interventions for children with ASD have historically emphasized in-toilet voiding outcomes using intensive schedules and reinforcement-based packages and may vary widely in components and reporting. Prior reviews indicate that, although many approaches can improve voiding outcomes, the evidence base remains dominated by small-n single-case experimental studies and heterogeneous outcome definitions, which limits comparability and confidence in generalizability [4,5]. Video modeling is often considered well suited for ASD because it reduces verbal demands and leverages visual learning, with evidence supporting its use across functional skill targets [13,14]. Toileting-focused studies have further shown that incorporating animation into video modeling can enable depiction of sensitive behaviors while retaining a complete routine sequence [19,20]. Our findings add to this literature by applying an animated stepwise routine in a mobile format designed for repeated use in naturalistic caregiver-managed settings.

A central conceptual clarification warranted by this study concerns the distinction between the digital platform and the behavioral intervention it delivered. The app served as a structured delivery medium for a multicomponent behavioral intervention that incorporated task analysis, behavior chaining, caregiver-mediated prompting, contingent token-based reinforcement, and stepwise animated video modeling. The study findings therefore reflect the preliminary feasibility and potential benefit of this intervention package as implemented through a caregiver-mediated digital platform rather than evidence on any single technological feature. This distinction is consistent with the broader literature on mHealth interventions, which emphasizes that behavioral mechanisms—not digital packaging—drive therapeutic change. Future work should use dismantling designs or component analyses to clarify the relative contributions of video modeling, token reinforcement, task analytic structure, and caregiver mediation to observed outcomes.

A central design decision was to operationalize toileting as a 16-step task analysis aligned with chaining principles. This allowed caregivers to target discrete steps and reinforce incremental progress rather than focusing exclusively on elimination outcomes [24,25]. Importantly, step completion was tracked even when elimination did not occur (eg, the child sat on the toilet but did not void), reflecting how toileting practice occurs in real-world routines. This may be relevant for children with ASD who often experience difficulty with sequencing and adaptive self-care skills [1]. The choice of animation (rather than live action) also addressed privacy and potential discomfort associated with human depictions of toileting-related behaviors while providing a consistent, child-friendly model.

Although the app included interactive elements, we interpret the motivational component primarily as reinforcement support. The token exchange rule (10 digital stickers exchanged for an immediate reward) implemented a clear contingency while allowing for individualized reinforcers (eg, in-app activities and/or caregiver-selected preferred items). This characterization avoids overstating “gamification” and makes the hypothesized mechanism explicit: repeated practice of a structured routine paired with contingent reward.

Beyond caregiver-recorded toileting logs, standardized assessment data provided descriptive contextual information that was generally consistent with the behavioral observations. On the K-SIB-R, toileting age-equivalent scores increased for all participants, and Personal Self-Care W scores moved closer to age-based expectations (Table 4). Norm-referenced deviation (SD units) decreased for each participant. These SD unit values represent distance from age norms rather than an effect size from a controlled comparison and should be interpreted cautiously. However, reporting a standardized measure alongside event-based and routine-based outcomes is consistent with the observation that the pattern of change was not limited to a single measurement approach.

This study has limitations consistent with early-stage digital health and single-case experimental research. First, the analytic sample was small (4 children), and 60% (6/10) of enrolled participants were not included due to discontinuation or insufficient exposure, underscoring the central importance of engagement and feasibility in caregiver-mediated digital interventions. Because baseline characteristics were not systematically available for all participants who were not included in the final analyses, formal comparison between completers and noncompleters was not possible; however, available contextual information and reasons for noncompletion or exclusion are summarized in Table S1 in Multimedia Appendix 1. Second, primary outcomes relied on caregiver-reported logs, which may introduce reporting bias and incomplete capture of events occurring outside caregiver supervision. Although caregivers received structured training and the research team reviewed records weekly with telephone follow-up when needed, independent observation and interobserver agreement data were not collected, limiting the reliability of caregiver-reported outcomes. Third, the follow-up phase consisted of 3 recording days at 3 months after the intervention, providing a limited sample of maintenance. Finally, day-level step completion percentages can be influenced by variation in the number of recorded events per day; therefore, trends over time are more interpretable than isolated daily values. In addition, technology-related factors may have affected feasibility. Children with limited interest in or tolerance for tablet-based activities may have difficulty engaging with the intervention, and caregiver time, digital literacy, and home technology environments may influence implementation. Therefore, app-based toileting interventions may not be appropriate for all children with ASD without additional engagement supports or caregiver assistance. Furthermore, because this was an early-stage single-case pilot feasibility study, a formal group-level sample size calculation was not performed. The enrollment target was determined pragmatically based on the intensive caregiver-mediated home implementation protocol, the multiple-baseline design, and anticipated recruitment constraints in young children with ASD requiring toileting support. These limitations indicate that the present findings should be interpreted as preliminary signals of feasibility and potential benefit rather than definitive evidence of effectiveness.

Future work should prioritize more rigorous evaluation, improved measurement, and strategies to sustain engagement while preserving the pragmatic, home-based nature of delivery. We have initiated an exploratory randomized controlled trial (target N=30) to evaluate effectiveness and implementation outcomes more robustly. This next phase can follow established reporting guidance for eHealth interventions and incorporate objective use metrics (in-app logs), a longer follow-up period, and independent outcome verification where feasible. Additional iterative development may also be warranted to support sustained engagement over longer periods given that low engagement was a reason for discontinuation in some families.

### Conclusions

A caregiver-implemented mobile app that integrates stepwise animated video modeling, task analysis–based chaining, and reinforcement support was feasible for families who engaged with the software and was associated with improvements in toileting outcomes and adaptive self-care indicators among young children with ASD. Larger controlled studies are needed to determine effectiveness, characterize engagement and attrition, and establish scalable implementation strategies.

## Supplementary material

10.2196/92147 Multimedia Appendix 1 Supplementary tables on treatment fidelity, participant noncompletion or exclusion, toileting methods, and exploratory outcomes.

## Abbreviations

ASD: autism spectrum disorder
BRIEF-P: Behavior Rating Inventory of Executive Function–Preschool Version
K-GARS-2: Korean version of the Gilliam Autism Rating Scale–Second Edition
K-PSI-4-SF: Korean Parenting Stress Index, Fourth Edition, Short Form
K-SIB-R: Korean Scales of Independent Behavior–Revised
mHealth: mobile health
NAP: nonoverlap of all pairs
