# Supplementary material for: A Stepwise Animated Video Modeling App With Token-Based Reinforcement for Toilet Training in Children With Autism Spectrum Disorder: Pilot Study Using Multiple-Baseline and Single-Case Designs
Source: JMIR Pediatr Parent. 2026 Sep 25;9:e92147. doi: 10.2196/92147 (PMC13614220; doi:10.2196/92147)
Supplement: Supplementary file 1 [file pediatrics-v9-e92147-s001.docx]

**Multimedia Appendix 1. Supplementary Tables**

This multimedia appendix provides supplementary tables referenced in the main manuscript. All values are reported descriptively.

**Table S1. Treatment fidelity checklist items**

Caregivers completed this 7-item checklist on five prespecified occasions during the intervention phase using a 5-point rating scale (1-5; higher indicates greater adherence). Checklist items were adapted from prior Korean studies (Lee, 2020).

| **Item** | **Checklist item** |
| --- | --- |
| 1 | Prepared the training environment. |
| 2 | Arranged training materials and reinforcers. |
| 3 | Explained the training to the child (eg, “It is time to go to the toilet”). |
| 4 | Guided the child to use the application for the toileting program. |
| 5 | Waited for the child to initiate/perform the target behavior. |
| 6 | Delivered reinforcement contingent on correct responding. |
| 7 | Responded appropriately to incorrect behavior  (eg, replayed the segment without reinforcement). |

**Table S2. Characteristics and Reasons for Noncompletion or Exclusion Among Participants Not Included in Final Analyses (N = 6).**

| **Participant** | **Analytic status** | **Age (y:m)** | **Sex** | **ASD diagnostic status** | **Training target** | **Baseline period (days)** | **K-GARS-2 autism index** | **Language level** | **Additional available contextual information** | **Reason for noncompletion or exclusion / Feasibility barrier** |
| --- | --- | --- | --- | --- | --- | --- | --- | --- | --- | --- |
| T01 | Excluded | 4:3 | Male | Confirmed | Seated urination | 13 | 118 (83rd %ile) | Single-syllable imitation only; no spontaneous speech | K-SIB-R, BRIEF-P, K-PSI-4-SF, device-use ability, and baseline toileting profile were available in the study summary file. | Post-enrollment ineligibility: ongoing ABA-based toilet training within 4 weeks of enrollment; toileting-related behavioral issues (playing with toilet water/lever), suggesting that problem behavior management may have been needed before training. |
| T02 | Discontinued | — | — | — | Defecation | — | — | — | Not available | Child condition: sleep disturbance worsened and the child's condition prevented consistent home-based toilet training. |
| T03 | Discontinued | — | — | — | Defecation | — | — | — | Not available | Low app/tablet engagement: the child showed no interest in and refused the tablet-based application provided for training. |
| T04 | Excluded | 3:10 | Female | Confirmed | Toilet sitting → seated urination | 4 (sitting); 5 (urination) | 96 (40th %ile) | Three-word imitation; no spontaneous speech | K-SIB-R, BRIEF-P, K-PSI-4-SF, device-use ability, and baseline toileting profile were available in the study summary file. | Toilet-sitting readiness/progression barrier: completed toilet-sitting training but did not progress sufficiently to the target urination/defecation training phase. |
| T07 | Excluded | — | — | — | Defecation | — | — | — | Not available | Caregiver recording nonadherence: caregiver did not complete observation logs reliably, resulting in insufficient data for analysis. Positive training effects were reported anecdotally (child began sitting on toilet and had occasional in-toilet defecation). |
| T08 | Excluded | 4:6 | Male | Confirmed | Standing urination | 18 | 79 (11th %ile) | Several spontaneous words (yes, no, mama, food) | K-SIB-R, BRIEF-P, K-PSI-4-SF, device-use ability, and baseline toileting profile were available in the study summary file. | Caregiver implementation and recording nonadherence: caregiver did not conduct training sessions and records were incomplete, with continued reliance on a child toilet and urination accidents noted. |

**Note.** ASD = autism spectrum disorder; ABA = applied behavior analysis; K-GARS-2 = Korean Gilliam Autism Rating Scale–Second Edition; K-SIB-R = Korean Scales of Independent Behavior–Revised; BRIEF-P = Behavior Rating Inventory of Executive Function–Preschool Version; K-PSI-4-SF = Korean Parenting Stress Index, Fourth Edition, Short Form. Age is reported in years:months at the time of enrollment. K-GARS-2 autism index values are reported as documented in the study summary file. Language level was documented by the research team based on caregiver report at enrollment. Baseline characteristics were not available in the study summary file for T02, T03, and T07; available information is limited to training target and reason for noncompletion or exclusion. Because baseline characteristics were not systematically available for all participants who were not included in the final analyses, formal comparison between completers and non-completers was not possible; available contextual information and reasons for noncompletion or exclusion are provided above.

**Table S3. Phase-level distributions of toileting method categories (n, %)**

Note: n indicates the number of recorded log entries with available toileting method coding in each phase.

| **Participant** | **Phase** | **n** | **Independent, n (%)** | **Communication /indication, n (%)** | **Caregiver prompting, n (%)** | **Accident (outside toilet), n (%)** |
| --- | --- | --- | --- | --- | --- | --- |
| T05 | Baseline | 10 | 0 (0.00) | 0 (0.00) | 0 (0.00) | 10 (100.00) |
|  | Intervention | 32 | 25 (78.13) | 0 (0.00) | 5 (15.63) | 2 (6.25) |
|  | Follow-up | 6 | 5 (83.33) | 0 (0.00) | 1 (16.67) | 0 (0.00) |
| T06 | Baseline | 42 | 13 (30.95) | 0 (0.00) | 2 (4.76) | 27 (64.29) |
|  | Intervention | 90 | 47 (52.22) | 1 (1.11) | 22 (24.44) | 20 (22.22) |
|  | Follow-up | 9 | 6 (66.67) | 0 (0.00) | 3 (33.33) | 0 (0.00) |
| T09 | Baseline | 51 | 0 (0.00) | 0 (0.00) | 40 (78.43) | 11 (21.57) |
|  | Intervention | 141 | 0 (0.00) | 15 (10.64) | 119(84.40) | 7 (4.96) |
|  | Follow-up | 14 | 0 (0.00) | 2 (14.29) | 12 (85.71) | 0 (0.00) |
| T10 | Baseline | 32 | 23 (71.88) | 3 (9.38) | 6 (18.75) | 0 (0.00) |
|  | Intervention | 144 | 101 (70.14) | 23 (15.97) | 12 (8.33) | 8 (5.56) |
|  | Follow-up | 14 | 7 (50.00) | 5 (35.71) | 2 (14.29) | 0 (0.00) |

**Table S4a. BRIEF-P pre- and post-intervention subscale scores (exploratory)**

Note: Scores are presented as BRIEF-P subscale scores as obtained from standardized scoring. Higher scores indicate greater executive function difficulties.

| **Participant** | **Timepoint** | **Emotional control** | **Working memory** | **Inhibitory control** | **Attention shifting** | **Plan /Organize** |
| --- | --- | --- | --- | --- | --- | --- |
| T05 | Pre | 25 | 44 | 42 | 24 | 24 |
|  | Post | 22 | 38 | 33 | 27 | 22 |
| T06 | Pre | 28 | 47 | 41 | 27 | 32 |
|  | Post | 21 | 43 | 41 | 22 | 22 |
| T09 | Pre | 24 | 39 | 24 | 19 | 22 |
|  | Post | 23 | 54 | 57 | 33 | 29 |
| T10 | Pre | 30 | 33 | 35 | 31 | 25 |
|  | Post | 22 | 49 | 45 | 25 | 24 |

**Table S4b. K-PSI-4-SF percentile ranks**

Note: Percentile ranks are reported for interpretability (higher percentile indicates greater parenting stress). Values reported as “>99” indicate above the 99th percentile.

| **Participant** | **Timepoint** | **Parental Distress** | **Parent-child Dysfunctional Interaction** | **Difficult Child** | **Total Stress** |
| --- | --- | --- | --- | --- | --- |
| T05 | Pre | 89.6 | 98.8 | 92.8 | 97.1 |
|  | Post | 89.6 | 87.5 | 75.5 | 89.1 |
| T06 | Pre | 30.2 | 69.9 | 94.6 | 70.9 |
|  | Post | 64.4 | 92.9 | 96.1 | 91.0 |
| T09 | Pre | 91.6 | >99 | >99 | >99 |
|  | Post | 89.6 | >99 | >99 | >99 |
| T10 | Pre | 93.5 | 97.4 | >99 | 98.8 |
|  | Post | 84.1 | 92.9 | >99 | 96.8 |

References

1. Lee S. Effects of video peer modeling intervention on symbolic play and social interaction in young children with autism spectrum disorder [master's thesis]. Ewha Womans University; 2020. Available from: <https://dspace.ewha.ac.kr/handle/2015.oak/254407>
